# Arthroplasty Utilization in the United States is Predicted by Age-Specific Population Groups

**DOI:** 10.5402/2012/185938

**Published:** 2012-10-23

**Authors:** Bronislava Bashinskaya, Ryan M. Zimmerman, Brian P. Walcott, Valentin Antoci

**Affiliations:** ^1^Department of Orthopedic Surgery, Massachusetts General Hospital, Harvard Medical School, Boston, MA 02114, USA; ^2^Boston University, Boston, MA 02215, USA; ^3^Department of Neurosurgery, Massachusetts General Hospital, Harvard Medical School, 55 Fruit Street, White Building Room 502, Boston, MA 02114, USA

## Abstract

Osteoarthritis is a common indication for hip and knee arthroplasty. An accurate assessment of current trends in healthcare utilization as they relate to arthroplasty may predict the needs of a growing elderly population in the United States. First, incidence data was queried from the United States Nationwide Inpatient Sample from 1993 to 2009. Patients undergoing total knee and hip arthroplasty were identified. Then, the United States Census Bureau was queried for population data from the same study period as well as to provide future projections. Arthroplasty followed linear regression models with the population group >64 years in both hip and knee groups. Projections for procedure incidence in the year 2050 based on these models were calculated to be 1,859,553 cases (hip) and 4,174,554 cases (knee). The need for hip and knee arthroplasty is expected to grow significantly in the upcoming years, given population growth predictions.

## 1. Introduction

As the post-World War II “baby boom” generation ages, a growing percentage of Americans will be living into their eighth decade and beyond [1]. Such demographic shift has significant implications for the design of a new healthcare delivery model [2, 3]. Medical conditions prevalent in the elderly are of particular interest and will have the greatest impact on the system. These include degenerative conditions, with severe arthritis afflicting over 15% of the population and estimated to surpass 20% (or 60 million people) by 2020 [4, 5]. As osteoarthritides of the knee and hip are known to increase with age, it is not surprising that the majority of knee and hip arthroplasty is performed in the elderly [6–12]. 

Being in a current state of healthcare reform with specific funding allocations being made, the understanding of future trends becomes critical. Considering that musculoskeletal complaints are the leading cause of medical claims, (with osteoarthritis encompassing the majority of disability in elderly adults), it is necessary to identify recent trends in arthroplasty and project future utilization needs. We hypothesize that the elderly subpopulation is correlated with arthroplasty utilization and can be used to predict arthroplasty utilization in the future. 

## 2. Methods/Materials

The data was analyzed anonymously, using publicly available secondary data; therefore no ethics statement is required for this work. To protect the confidentiality of patients, the dataset suppressed reporting when values were based on 10 or fewer discharges or when fewer than two hospitals in the state were reporting. Incidence data from the US Nationwide Inpatient Sample was queried from 1993 to 2009 (the most recent available year). Weighted national estimates were provided from the Agency for Healthcare Research and Quality (AHRQ), Healthcare Cost and Utilization Project's Nationwide Inpatient Sample (NIS), based on data collected by individual states and provided to the AHRQ. The total number of weighted discharges in the USA is based on the NIS total of =39,434,956. Statistics based on estimates with a relative standard error (standard error/weighted estimate) greater than 0.30 were excluded. Statistics were only based on hospitals that meet the definition of  “community hospital”—nonfederal, short-term, general, and other specialty hospitals, including public hospitals and academic medical centers. Federal, rehabilitation, and psychiatric hospitals, as well as alcoholism/chemical dependency treatment facilities were excluded from analysis.

The principal procedure was defined as the definitive treatment during the hospital admission (not diagnostic or exploratory). The unit of analysis was discharge: if a particular procedure occurred multiple times during the same admission, it was only counted once. Knee and hip arthroplasty were identified as principal procedures using clinical classifications software (CCS) of ICD-9-CM codes 152 and 153, respectively, [13] (Table 1). Information regarding incidence, combining both primary and revision procedures, was extracted. The United States Census Bureau was queried for population data from the same study period [14] and also used to provide future projections [15]. 

Statistical analysis was performed using the R programming environment version 2.15.1 (R Core Team (2012). R: a language and environment for statistical computing. R Foundation for Statistical Computing, Vienna, Austria. ISBN 3-900051-07-0, URL http://www.R-project.org/). To test our first hypothesis, we identified three population groups based on available United States Census data: total population, population over 64 years of age, and population over 84 years of age. These values were then used to generate individual scatterplots to allow for a visual interpretation between the variables. A linear relationship was assumed based on the distribution of the plotted variables. A Pearson product-moment correlation coefficient was then calculated for each of the three population groups to determine the strength of association. 

To analyze the ability of population trends to predict future arthroplasty incidence, linear regression analysis, fitted using the least squares approach, was utilized. Models were created for each of the various population groups to extract individual regression coefficients and *R*-squared values. An analysis of variance table was used to compare the different models. Visual regression diagnostics were performed by plotting residual versus fitted values, standardized residuals versus theoretical quantiles, square root of standardized residuals versus fitted values, and standardized residuals versus leverage. Finally, using the regression formula, arthroplasty trends in future years were predicted using estimated population data from the United States Census Bureau [15]. 

## 3. Results

**Figure 1:**
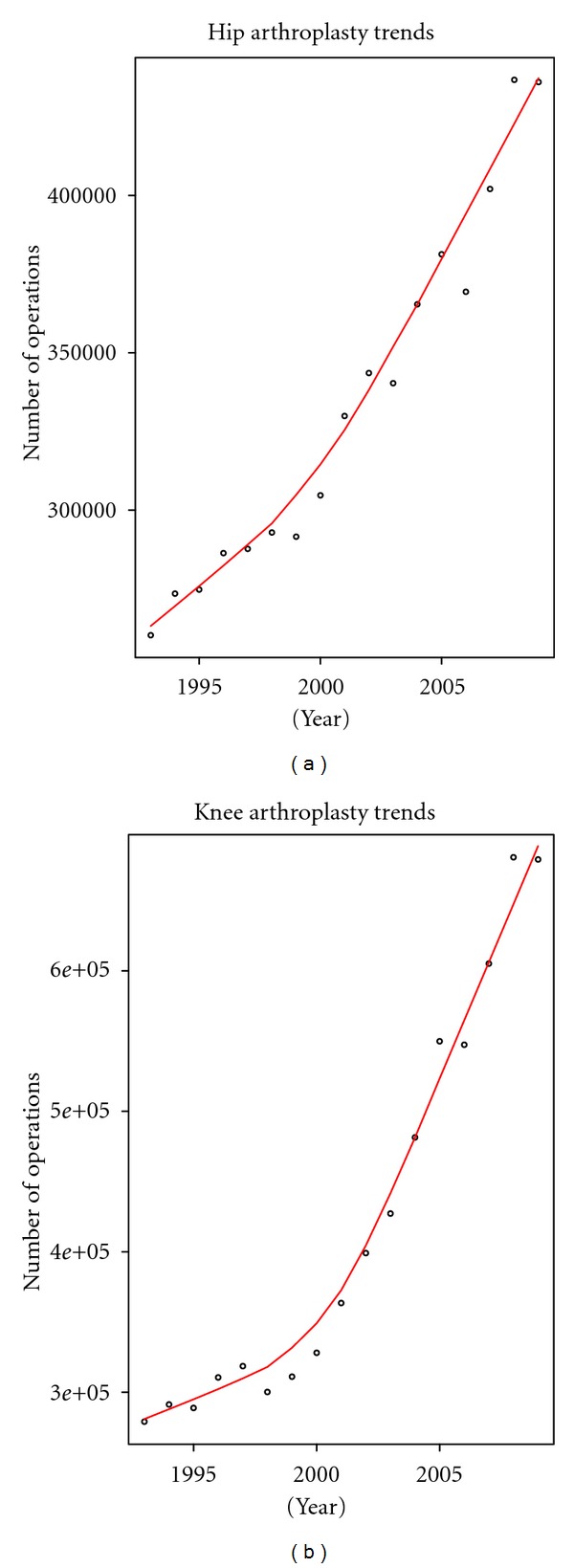
Hip and knee arthroplasty trends over time. Hips and knee arthroplasty incidence has risen in a nonlinear fashion over time. The smoothed scatterplot trend line represents a locally weighted polynomial regression.

**Figure 2:**
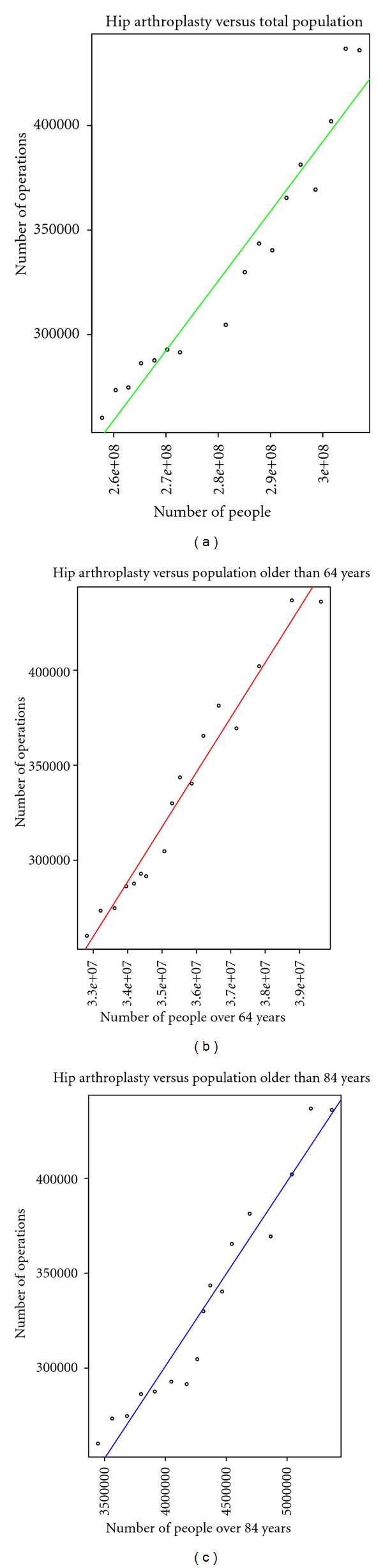
Hip arthroplasty versus population groups. Hip arthroplasty can be predicted by linear regression using the total population ((a)—green regression line), only the population over 64 years of age ((b)—red regression line), and only the population over 84 years of age ((c)—blue regression line).

**Figure 3:**
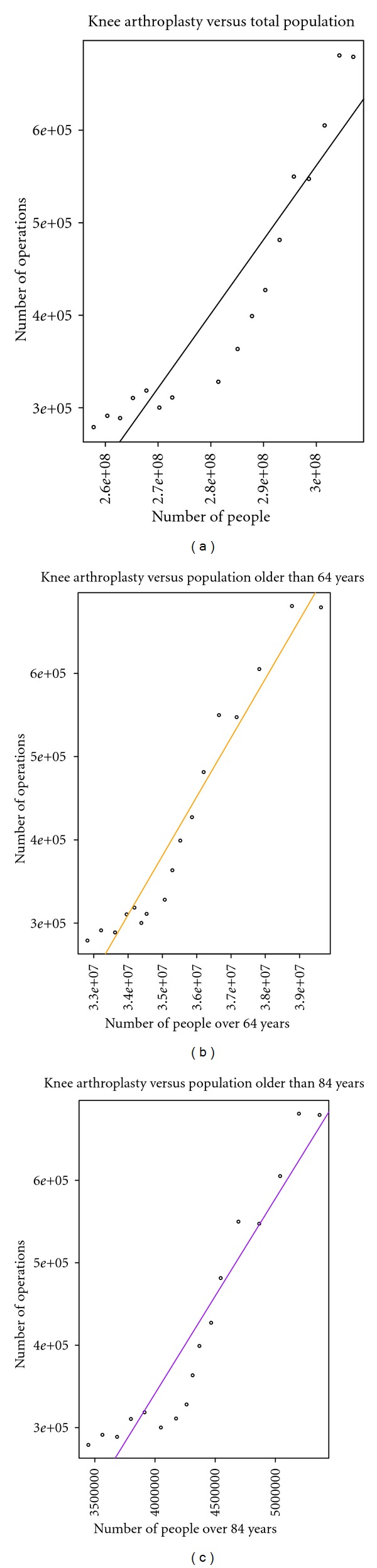
Knee arthroplasty versus population groups. Knee arthroplasty can be predicted by linear regression using the total population ((a)—black regression line), only the population over 64 years of age ((b)—orange regression line), and only the population over 84 years of age ((c)—purple regression line).

17 years of data (1993–2009) were available in the Nationwide Inpatient Sample, providing incidence data for hip and knee arthroplasty. The incidence of hip arthroplasty ranged from 260,200 to 436,700 cases per year (median 329,900 ± standard error 13773). The incidence of knee arthroplasty ranged from 279,101 to 680,839 (median 363,536 ± standard error 34,330) (Figure 1). Scatterplots were also generated assuming a linear relationship between procedure incidence and the three population groups (Figures 2 and 3). A Pearson product-moment correlation coefficient matrix revealed the strongest association between arthroplasty (both hip and knee) and population subgroup “greater than 64 years of age” (Table 2). 

Linear regression models were then created for each of the various age groups and the incidence of operative procedures (Figures 1 and 2). The *X* value of “population > 64 years” was the most accurate model for both hip arthroplasty and knee arthroplasty based on *R*-squared analysis (Table 3). Arthroplasty followed a linear model with the population group > 64 years in both hip and knee groups; *R* ^2^ = 0.969 and 0.944, respectively. An analysis of variance table revealed that “population > 64 years” consistently resulted in the smallest residual sum of squares in both hip and knee regression models when compared to other *X* variables (Table 4). Visual regression diagnostics were performed by plotting residual versus fitted values, standardized residuals versus theoretical quantiles, square root of standardized residuals versus fitted values, and standardized residuals versus leverage confirmed that a linear regression analysis was appropriate. Using the regression formula, arthroplasty trends in future years were predicted using estimated population data from the United States Census Bureau (Table 5). Projections for procedure incidence in the year 2050 based on these models were calculated to be 1,859,553 cases (hip) and 4,174,554 cases (knee).

## 4. Discussion

The population of the United States is growing rapidly, with the proportion of elderly citizens projected to grow even faster as the baby-boomer generation ages. With age, a cumulative “wear and tear” summates at the cellular, organ and population based levels [16–21]. Osteoarthritis is one of the major diseases predicted to expand in this population, along with cancer [22], pneumonia [23], and heart disease [24, 25]. Joint arthroplasty is one surrogate of advanced osteoarthritis and has been projected to occupy a significant portion of the healthcare expenditure in the next couple decades. Previous investigations have predicted an increase in total joint arthroplasty demand [26]. In a study by Kurtz et al., projections for hip arthroplasty were expected to grow over a 25-year period, with a 174% and 673% estimated increase in hip and knee arthroplasty, respectively [26]. We confirm those trends using different methods, primarily taking into account the growing aging population, in order to better predict resource utilization. Complementary studies have assessed historical trends in arthroplasty utilization using different patient populations, which is useful in anticipating future needs [27]. In a study of only Medicare enrollees (≥65 years of age) by Cram et al., knee arthroplasty volume increased to a similar extent with the findings of our study [27]. 

Many additional factors must be considered in conjunction with age when determining the impact of degenerative musculoskeletal conditions on the healthcare systems. Environmental (patient-centered) factors, such as metabolic or inflammatory conditions, contribute significantly to the incidence of osteoarthritis of the weight bearing joints [28, 29]. Therefore, the increasing incidence of hip and knee arthroplasty is more complex than a direct relationship with the proportion of elderly in the population. This holds particularly true in a population where obesity has reached epidemic proportions [30–32]. The combined (and possible synergistic) effect of age, weight, and patient specific factors is likely to account for the overall increase in the incidence of arthroplasty, although this has not yet been rigorously studied. 

While genetic predisposition for osteoarthritis exists and we cannot avoid the inherent mechanical stresses of bipedalism [33–38], straightforward interventions have been demonstrated to be effective in tertiary prevention of osteoarthritis and can be extrapolated to primary and secondary prevention as well. Specifically, weight loss reduces symptomatic knee osteoarthritis [39]. Additionally, either aerobic or resistance exercise regimens are effective in improving measures of disability, physical performance, and pain associated with osteoarthritis in older persons [40]. Given the major impact on the national healthcare “bill” and local resource utilization in individual hospitals, improved efforts directed at preventative care are warranted [36, 41]. Indeed, modification of factors that contribute to symptomatic osteoarthritis, such as obesity, and optimization of other interventions, such as injectables or rehabilitation regimens, may modify the predicted growth in arthroplasty demand.

The main strength of this study includes the use of a well-established national database. Millions of patients are analyzed in a standardized manner over a longitudinal period. Limitations include the national unit of analysis modeling that makes regional differences difficult to account for and that may be influenced by differential reimbursement patterns or socioeconomic factors [42, 43]. It is also impossible to anticipate the effect of medical innovations, scientific discovery, or clinical research that could dramatically alter procedure incidence and result in deviation from the calculated projection model. For example, with the discovery of *H. pylori* as the causative organism for the majority of foregut ulcers, targeted medical therapy has led to a significant decrease in surgical procedures associated with the same [44]. Alternatively, unforeseen clinical efficacy established by reputable clinical trials can lead to changes in surgical procedure incidence [45]. Also to consider are the burgeoning ranks of patients undergoing primary arthroplasty that may portend a dramatic rise in individuals requiring revision arthroplasty, costly procedures not able to be modeled for in this study [46, 47]. Indications for arthroplasty are dynamic, which limits our accuracy in predicting future surgical volume, although prior studies on this subject have similarly projected arthroplasty growth [26]. 

This database does not contain information on the degree of severity of osteoarthritis and does not capture other clinical data points such as ancillary treatments, patient weight, mobility status, or time interval from symptom onset to treatment. As with all data recorded in national databases, various coding anomalies are known to exist [48]. We attempted to eliminate this bias by focusing exclusively on the principal diagnosis (i.e., the major determinant of reimbursement rates) with the assistance of CCS grouping that systematically and comprehensively identifies key ICD-9-CM procedure codes. 

## 5. Conclusion

The demand for knee and hip arthroplasty is expected to rise significantly, given recent trends in practice and predicted increase in the elderly population. Resource allocation and surgeon training should prepare physicians to serve these anticipated needs. 

## Figures and Tables

**Table 1:** ICD-9-CM codes grouped according to clinical classifications software.

CCS Code	Procedure	ICD-9-CM Codes
152	Knee arthroplasty	0080; 0081; 0082; 0083; 0084; 8141; 8142; 8143; 8144; 8146; 8147; 8154; 8155

153	Hip arthroplasty	0070; 0071; 0072; 0073; 0074; 0075; 0076; 0077; 0085; 0086; 0087; 8151; 8152; 8153; 8169

**Table 2:** Pearson product-moment correlations.

	Hip	Knee
Population total	0.9660183 (95% CI 0.9060745, 0.9879475)	0.9318893 (95% CI 0.8173353, 0.9755691)
Population > 64 years	0.9844568* (95% CI 0.9563160, 0.9945204)	0.9715806* (95% CI 0.9210546, 0.9899387)
Population > 84 years	0.9723646 (95% CI 0.9231783, 0.9902188)	0.9489339 (95% CI 0.8609842, 0.9817860)

*The strongest correlation and the narrowest 95% confidence intervals (CI).

**Table:** (a)

*X*	Coefficient	Intercept	*R*-squared
Population total	3.333*e* − 03± 2.302*e* − 04	−6.074*e* + 05± 6.514*e* + 04	0.933
Population > 64 years	2.880*e* − 02± 1.327*e* − 03	−6.906*e* + 05± 4.726*e* + 04	0.969
Population > 84 years	9.722*e* − 02± 6.027*e* − 03	−8.784*e* + 04± 2.636*e* + 04	0.945

±Standard error.

**Table:** (b)

*X*	Coefficient	Intercept	*R*-squared
Population total	8.013*e* − 03± 8.053*e* − 04	−1.842*e* + 06± 2.278*e* + 05	0.868
Population > 64 years	7.085*e* − 02± 4.457*e* − 03	−2.099*e* + 06± 1.588*e* + 05	0.944
Population > 84 years	2.365*e* − 01± 2.030*e* − 02	−6.046*e* + 05± 8.876*e* + 04	0.901

±Standard error.

**Table:** (a)

*X*	Degrees of freedom	Residual sum of squares
Population total	15	3,447,435,118
Population > 64 years	15	1,591,638,084
Population > 84 years	15	2,812,651,855

**Table:** (b)

*X*	Degrees of freedom	Residual sum of squares
Population total	15	4.2181*e* + 10
Population > 64 years	15	1.7962*e* + 10
Population > 84 years	15	3.1904*e* + 10

**Table 5:** Prediction of future arthroplasty utilization.

Year	Population > 64 years (projected)	Hip arthroplasty	Knee arthroplasty
2010	40,229,000	467,995	751,224
2015	46,837,000	658,305	1,219,401
2020	54,804,000	887,755	1,783,863
2025	63,907,000	1,149,921	2,428,810
2030	72,092,000	1,385,649	3,008,718
2035	77,543,000	1,542,638	3,394,921
2040	81,238,000	1,649,054	3,656,712
2045	84,456,000	1,741,732	3,884,707
2050	88,547,000	1,859,553	4,174,554
